# Ontology of physics for biology: representing physical dependencies as a basis for biological processes

**DOI:** 10.1186/2041-1480-4-41

**Published:** 2013-12-02

**Authors:** Daniel L Cook, Maxwell L Neal, Fred L Bookstein, John H Gennari

**Affiliations:** 1 Department of Physiology & Biophysics, University of Washington, Seattle 98195, USA; 2 Department of Biomedical & Health Informatics, University of Washington, Seattle 98195, USA; 3 Department of Bioengineering, University of Washington, Seattle 98195, USA; 4 Department of Statistics, University of Washington, Seattle 98195, USA

## Abstract

**Background:**

In prior work, we presented the Ontology of Physics for Biology (OPB) as a computational ontology for use in the annotation and representations of biophysical knowledge encoded in repositories of physics-based biosimulation models. We introduced OPB:*Physical entity* and OPB:*Physical property* classes that extend available spatiotemporal representations of physical entities and processes to explicitly represent the thermodynamics and dynamics of physiological processes. Our utilitarian, long-term aim is to develop computational tools for creating and querying formalized physiological knowledge for use by multiscale “physiome” projects such as the EU’s Virtual Physiological Human (VPH) and NIH’s Virtual Physiological Rat (VPR).

**Results:**

Here we describe the OPB:*Physical dependency* taxonomy of classes that represent of the laws of classical physics that are the “rules” by which physical properties of physical entities change during occurrences of physical processes. For example, the fluid analog of Ohm’s law (as for electric currents) is used to describe how a blood flow rate depends on a blood pressure gradient. Hooke’s law (as in elastic deformations of springs) is used to describe how an increase in vascular volume increases blood pressure. We classify such dependencies according to the flow, transformation, and storage of thermodynamic energy that occurs during processes governed by the dependencies.

**Conclusions:**

We have developed the OPB and annotation methods to represent the meaning—the biophysical semantics—of the mathematical statements of physiological analysis and the biophysical content of models and datasets. Here we describe and discuss our approach to an ontological representation of physical laws (as dependencies) and properties as encoded for the mathematical analysis of biophysical processes.

## Background and aims

Physiological knowledge is based on physically observable properties of biological entities and how those properties change values during biological processes. The parsing of biological function into physical entities and processes is fundamental to how we observe, represent, and analyze biology using a range of expressions from the purely descriptive (e.g., “increased blood pressure increases blood flow”) to formal quantitative mathematical expressions (e.g., a pressure gradient is related to blood flow via the fluid version of Ohm’s law). Whether described and illustrated in textbooks of physiology or formalized in complex, rigorous mathematical biosimulation models, the semantics of physiological processes and their dependence on thermodynamics are generally implicit in the representations.

In prior work, we described how the OPB extends and adapts classes from the Basic Formal Ontology (BFO) [1] and the General Formal Ontology (GFO) [2] to represent OPB:*Physical entity* and OPB:*Physical property* classes [3,4] that extend BFO and GFO spatiotemporal representations of physical entities and processes to explicitly represent the thermodynamics and dynamics of physiological processes. Here we take the next step by describing OPB:*Physical dependency* classes as formal representations of the laws of classical physics that are the “rules” by which physical properties of physical enities change during occurrences of physical processes. For example, the fluid analog of Ohm’s law (as in electric currents) is used to describe how a blood flow rate depends on a blood pressure gradient. Hooke’s law (as in elastic deformations of springs) is used to describe how an increase in vascular volume increases blood pressure.

Our very utilitarian, long-term aim is to develop computational tools for creating and querying formalized physiological knowledge [5] for use by multiscale “physiome” projects such as the EU’s Virtual Physiological Human (VPH) and NIH’s Virtual Physiological Rat (VPR). A knowledge representation problem common to such projects is that knowledge must be shared between domain-specific “silos” that employ different data formats and computational languages. Expressed most simply, our working hypothesis is that an ontological formalization of the mathematical language of classical physics can provide a syntax and semantics for logically representing the biological content of datasets and analytical models according to their biophysical meanings better than *ad hoc* documentation and local naming/coding schemata. Thus, our goal for the OPB is to formally represent the “biophysical semantics” of the mathematical statements of physiological analysis and simulation to formally map and query the biophysical content of models and datasets. As example use-cases, we are developing and testing this approach in the domains of cardiovascular physiology [6,7] and systems biology [8] using our SemGen software and knowledge structures [9] to cast systemic and cellular physiology models into a prototype semantic human physiome [5].

We have successfully built on this premise by developing OPB-based computational tools and a workflow with which we annotate, aggregate, and query [8,10,11] the biophysical content of available physiological models written in the JSim language [12] (available from the National Simulation Resource [13]), models written in CellML [14] (available from PMR2 model repository [15]), models written in SBML [16] (available from the BioModels Database [17]), and BioPAX pathway data [18]. We are currently testing these tools in the context of the VPR multiscale physiological modeling project [6]. These tools have used only the OPB:*Physical property* classes [3] to create composite annotations [9,19,20] to annotate data and variables (e.g., blood pressure and flow rate). However, if we annotate only physical properties of participating entities (e.g., things such as hearts and portions of blood), then we ignore the dependencies amongst property values (e.g., physical laws and axioms) by which physical processes occur. Thus, we have now extended OPB to represent a taxonomy of such physical dependencies by which property values change during processes as a first step toward representing a taxonomy of physical processes.

We anticipate that the hierarchy of logically-defined OPB:*Physical dependency* classes we present here will accelerate physiome-level modeling efforts in several ways. First, it will help automate the cumbersome model annotation process. Furthermore, OPB:*Physical dependency* annotations will communicate valuable information about the assumptions underlying a model’s mathematical structure. This is crucial within the context of community-level model reuse because, when repurposing a model, modelers must determine whether its underlying assumptions preclude its use for a modeling project. Incorporating dependency annotations into models also provides a basis for performing qualitative, up/down perturbation experiments on the modeled system without the need for a numerical solver (see “Discussion and next steps”).

We extend our prior work in semantic annotation to not only annotate model variables but also computations amongst variables (i.e. the equations) against OPB:*Physical dependency* classes that semantically represent the meaning of the computations in terms of biophysical systems dynamics. For example, a model that simulates blood flow will, typically, calculate the dependence of blood flow rates (OPB:*Fluid flow rate*) on blood pressure differentials (OPB:*Fluid pressure*) along flow paths using, in the simplest case, a fluid analogue of Ohm’s law (OPB:*Resistive flow dependency* > OPB:*Fluid flow dependency*). Similarly, that model will typically calculate blood pressures as functions of the volumes of blood in a vessels according to fluid analogues of Hooke’s law (OPB:*Capacitive force dependency* > OPB:*Fluid capacitive dependency*).

**Figure 1:**
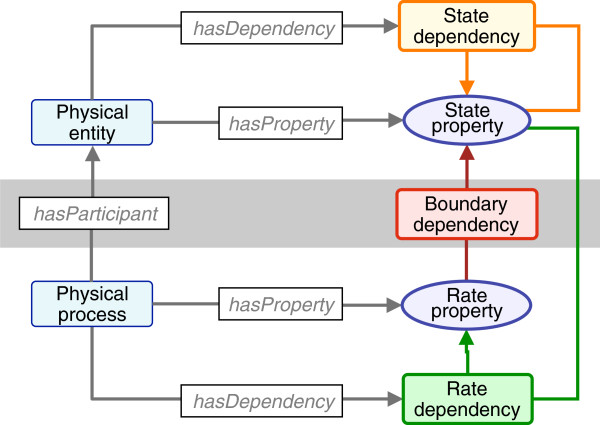
**OPB classes represent *physical entities *and *processes *(blue icons) that have *physical properties *(purple) which are players (blue arrows) in biophysical *dependencies *that represent biophysical mathematical computations.***Continuants* are above the gray “boundary” bar; *processural entities*, below. Spatiotemporal categories are at left, biophysical classes at right.

**Figure 2:**
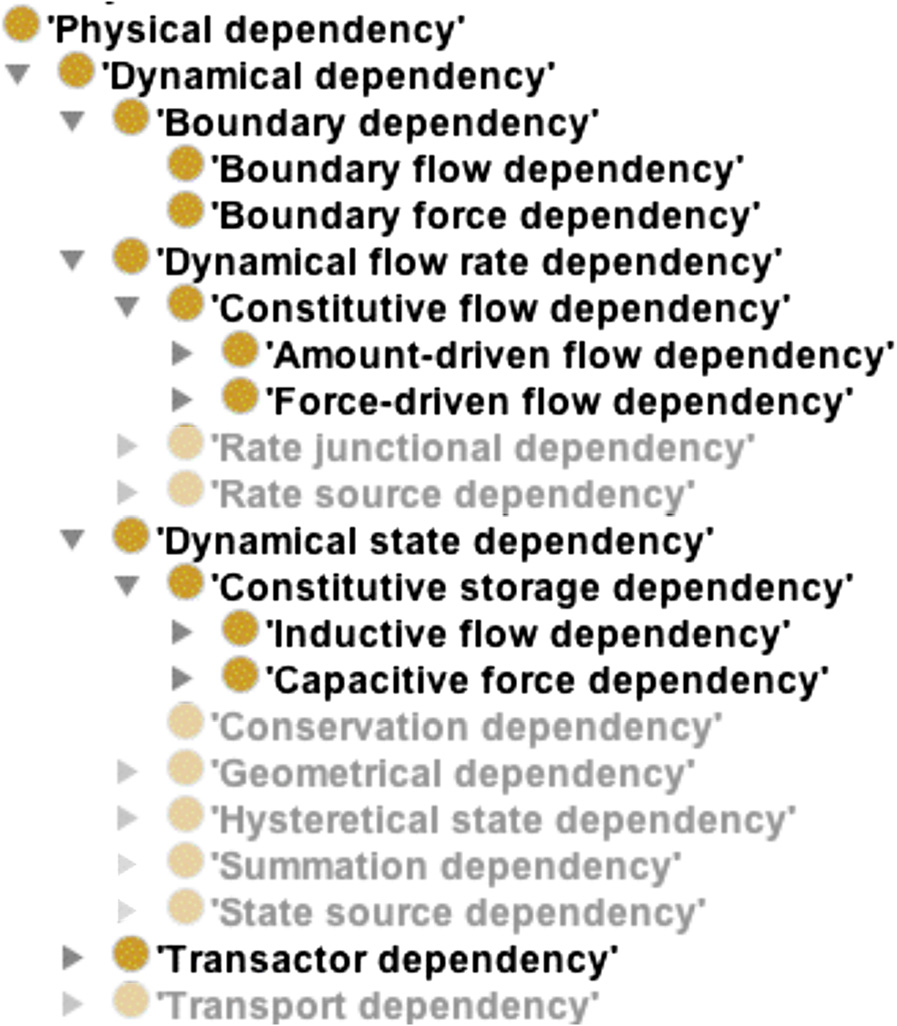
**OPB:** ***Physical dependency *** **class taxonomy showing selected OPB:** ***Dynamical dependency *** **classes as discussed in this paper; see the OPB.owl file for others.**

**Figure 3:**
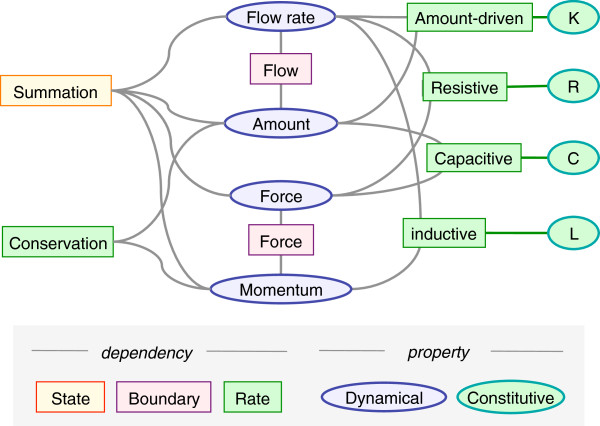
**Schematic map that expands on Figure**1**to show OPB:*hasPropertyPlayer *relations (gray lines) between key OPB:*Dynamical dependencies *classes as rectangles for OPB: *Dynamical state dependency *, OPB: *Boundary dependency *, and OPB: *Dynamical flow rate dependency *.** OPB:*Physical property* players in these dependencies are shown as blue ovals for OPB:*Dynamical properties* and gray ovals for OPB:*Constitutive properties*. K, R, C, and L constitutive properties are, respectively: OPB:*Reaction rate constant*, OPB:*Resistance*, OPB:*Capacitance*, and OPB:*Inductance.*

Thus, the specific goals of the current effort are to: (1) define the OPB:*Physical dependency* class to represent the various quantitative dependencies between values of OPB:*Physical properties* (as in Figure 1), (2) classify dependencies according to a conceptual framework based on system dynamical theory (Figures 2, 3), and (3) subclass the dependencies to apply to single and multiple biophysical domains (OPB:*Physical domain*; e.g., fluid flow, chemical kinetics; Table 1). In future work, we propose to map OPB:*Physical dependency* classes to corresponding OPB:*Physical process* classes as a basis for creating a semantic map of the human physiome built from the physiological knowledge extracted semiautomatically from available biosimulation models as “SemSim” models [7] and integrated as extended “PhysioMaps” [5,10].

**Table 1:** **Examples of OPB:** ***Dynamical dependency *** **classes with property players and corresponding constitutive proportionality classes**

**Dynamical dependency**			**Rate property player**	**Other property player(s)**	**Constitutive proportionality**
Constitutive flow dependency					
	Amount-driven flow dependency		Flow-rate	∆ Amount	Resistance
	Force-driven flow dependency		Flow-rate	∆ Force	Rate constant
		Resistive flow dependency	Flow-rate	“	Resistance
		Coupled flow dependency	Flow-rates	“	Modulus
Constitutive storage dependency					
	Capacitive dependency		∆ Force	Amount	Capacitance
	Inductive dependency		∆ Force	Momentum	Inductance
Transactor dependency			Either	Any	Coefficient

## Approach and scope

OPB is curated in the web ontology language (OWL [21]) using the Protege-OWL [22] ontology editor. OPB is available from BioPortal [23]. Currently, OPB encompasses the physics of discrete entities that can be analyzed with algebraic or ordinary differential equations (ODE); we have deferred work on the representation of continuum physics requiring partial differential equations (PDE) of spatial gradients. As a domain strategy, we base the OPB on theories of classical physics as expressed in texts of basic, classical physics and as applied to physiological systems including biomechanics (e.g., [24]), electrophysiology (e.g., [25]), chemical biophysics (e.g., [26]), and large-scale physiological integration (e.g., [27]).

As a curatorial strategy, we base our representation on an extensive range of use-cases encountered in our project to establish a “semantic physiome” that consists of “semantic simulation” (SemSim) models [28] derived by semiautomatically parsing and annotating biosimulation models as available from model repositories in a variety of computational languages. These sources afford us a wide range of use-cases that span multiple structural scales and biophysical domains with challenging abstractions.

We generalize engineering-oriented approaches to represent multiscale, multidomain physiological processes as a useful knowledge tool that leverages recent progress in computational biomedical ontology [5]. Thus our goals and approaches differ from other ontological approaches to physical phenomena such as naive physics as developed for artificial intelligence applications [29], the representation of the mathematics of engineering analysis [30], or strictly spatiotemporal representations of entities and events as articulated by Dorato et al. [31].

## Foundational representation of biophysics

Using the representational schema illustrated in Figure 1, the OPB is designed to express the physical intuition that, at any instant in time, any *physical entity* (e.g., a portion of blood, cell) exists in a *physical state* that is defined, in part, by the values of its *state properties* (e.g., fluid volume, cell location). State property values change during physical processes (e.g., blood volumes change as blood flows, bones move as forces are applied) at *process rates* (e.g., blood flow rate, bone velocity) determined, in turn, by the state properties of the entities that participate in the process. This merely restates the Laplacian precept that future physical states are fully determined by current physical states. Our aim is not to author a formal model of the “real” world as a kind of physical “realism” but to produce a semantic model of physical concepts as they are used by biophysicists, bioengineers, and physiologists to analyze and model the “real” world. Thus, OPB should be capable of representing statements of physics that concern such “unreal” entities as frictionless planes, inviscid blood or spherical hearts.

As a first step, we adopt the foundational distinction between physical entities (“continuants”) and the physical processes (“occurrents”) by which the entities change. Whereas BFO represents the spatiotemporal aspects of reality, OPB represents the complementary and orthogonal thermodynamic and dynamical aspects of the same reality. Thus OPB:*Physical entities* participate (via the *hasParticipant* relation) in OPB:*Physical processes* wherein thermodynamic energy flows amongst participants (see below). Entity and process classes are attributed with physical properties (via the *hasProperty* relation) that are classified as, respectively, OPB:*Dynamical state properties* and OPB:*Dynamical rate properties*, so-named because each determines, respectively, the states and rates of change of the thermodynamic energy content of participating physical entities (see OPB:*Thermodynamic dependency*, below). The values of these properties depend upon one another according to the laws of physics, expressed as mathematical relations and used to compute the quantitative implications of biophysical hypotheses.

In prior work [3], we introduced the OPB:*Physical property* taxonomy. Here we extend our representation to OPB:*Physical dependency entity* classes (Figure 1) whose main subclass, OPB:*Dynamical dependency*, is defined as “…a mathematical relationship by which the values of dynamical properties of entities that are role players in the dependency determine the flow or distribution of energy amongst physical entities that are bearers of the properties”. That is, physical dependencies encompass the “rules” by which physical entities property values change and energy flows during occurrences of physical processes. As such, OPB:*Physical dependency entity* may qualify a subclass of GFO: *Concept* (i.e., “categories that are expressed by linguistic expressions and which are represented as meanings in someone’s mind” [32]). However, as the top class in the OPB is defined as “formal abstraction of the real world created for the science of classical physics for describing and analyzing real physical entities and processes”, the OPB would seem to be apart from the BFO representation of “reality” [33].

### System dynamical analogies span biophysical domains

The OPB representational schema illustrated in Figure 3 is based on well-developed theories of system dynamics [34-37] that recognize and represent analogies between physical phenomena in different physical domains (OPB:*Physical domain*). For example, Ohm’s law, originally derived for modeling electrical current flow, describes analogous relations for viscous resistance to fluid flow and mechanical translation. Similarly, Hooke’s law derived for elastic spring compression applies to electrical capacitors and extensible fluid vessels. Following Borst et al. [34], we have formalized such analogies for classifying physical properties (e.g., fluid pressure, chemical concentration) [38] and here present a taxonomy of the physical laws that are the basis for the analogies. For example, OPB:*Resistive flow dependencies* (“Resistive” in the figure) are analogues of Ohm’s law that relate a flow rate (classically, electrical current in amperes) to a force (an electrical potential difference, in volts) and a resistive constitutive property (typically a “resistance” parameter in Ohms). OPB:*Capacitive force dependencies* (“Capacitive” in the figure) are analogues of Hooke’s law that relate an amount (e.g., the volume of fluid in an elastic vessel) to a force (e.g., the fluid pressure in the vessel) across multiple dynamical domains. We have formalized and extended these dynamical analogies from the engineering domain to the domains of physiological processes.

## Physical dependencies represent physical laws

Physical dependencies represent fundamental rules by which material, electrical charge, and thermodynamic energy are transfered and stored in and amongst participants in physical processes. Thus, an OPB:*Physical dependency* “…is an axiom, definition, or empirical law of classical physics that relates the values of physical properties of physical entities and processes to one another”. For example, the area of a circle depends on its diameter; electrical current flow in a wire depends on its voltage differential and a resistance to flow; the change of fluid volume in a vessel depends on net fluid inflow and outflow rates and the elasticity of the vessel. OPB:*Physical dependencies* logically represent the quantitative relationships between the values of physical properties of the physical entities that participate in physical processes. In parallel with the RO:*has_participant* relationships [39] of entities to procsses as in Figure 1, OPB defines the OPB:*hasPropertyPlayer* (blue arrows in Figure 1) that relate physical properties to the dependencies that describe how the values of the properties depend upon one another.

Thus, for example, a dependency representing Ohm’s law would have as property players a current (I), a voltage differential (E), and a resistance (R). Just as process participants must be distinguished according to their participatory role (RO:*has_participant*), players and their roles in dependencies are related by OPB:*hasPropertyPlayer* object relations. For example, I, E, and R are related by OPB:*hasFlowPlayer,* OPB:*hasForcePlayer,* and OPB:*hasConstitutiveProportionality* for a representation of Ohm’s law where “Flow”, “Force” and “Constitutive” refer to OPB superclasses for electrical current, voltage, and resistance, respectively [3]. We will describe selected examples of three OPB:*Dynamical dependencies* classes: OPB:*Boundary dependency*, OPB:*Dynamical state dependency*, and OPB:*Dynamical flow rate dependency* as shown in the class taxonomy in Figure 2.

### Boundary dependencies

A boundary dependency represents how a change in an *amount* or *momentum* (i.e., a *state property*) of a physical entity depends on a *flow rate* or *force* that traverses or impinges on the boundary of the entity over a span of time. For example, blood flows across the boundary from one portion of fluid (OPB:*Portion of fluid*, e.g., blood in the proximal aorta) to another (e.g., blood in the distal aorta) reducing and increasing, respectively, the *amount* of blood in each portion. This basic principle, an application of “Stokes theorem” to discrete systems, is represented by subclasses of OPB:*Boundary flow dependency* which holds for the flow of conserved quantities in multiple biophysical domains such as mass, charge, and energy flows across entity boundaries. For discrete entities, this dependency takes the mathematical form, ∆*volume = *∫*(volume_flow_rate) dt*, or in the derivative form (*d(volume)/dt = volume_flow_rate*). These mathematical relations simply state that the change in amount of “stuff” (e.g., volume of blood; OPB:*Fluid volume*) in an entity changes over a span of time as the temporal integral of the flow rate of stuff (e.g., volume flow rate of blood; OPB:*Fluid flow rate*). This dependency applies irrespective of the structural constitution and material properties of the participating entities and depends solely on the fluxes across the boundary (OPB:*Physical boundary*) between entities.

The OPB:*Boundary force dependency* is an analogous, but less familiar, dependency that represents a change in the value of a momentum state property (OPB:*Momentum property*) as the temporal integral of a force (OPB:*Force property*) over a span of time. For example, the longer a force is applied to a ball, the faster the ball will roll; i.e., the more momentum it will have. When temporally differentiated, this dependency is a restatement of Newton’s Second Law (i.e., that force is a product of mass and acceleration; f = ma).

### State dependencies

According to boundary dependencies, flows or forces acting across the boundary of a physical entity necessarily change the entity’s amounts or momenta, respectively, which are changes of physical state (OPB:*Physical state*) that may change other state properties. For example, the net flow of blood into a vessel changes the vessel’s volume (OPB:*Fluid volume*) which changes values of other spatial properties such as the vessel’s length or diameter. Such dependencies amongst an entity’s state properties are represented as subclasses of OPB:*Dynamical state dependency* that determines “…how attributes of a physical entity are related at a temporal instant” and thus represent static relationships amongst entity state properties. In addition to OPB:*Spatial dependency* (as in our vascualar example), OPB:*Dynamical state dependency* also includes the subclass OPB:*Summation dependency*, which applies to multiscale entities and, for example, accounts for how, at any instant, the volume of an entity is the sum of the volumes of its proper parts.

### Rate dependencies

OPB:*Dynamical flow rate dependencies* represent a broad variety of biophysical mechanisms by which physical entities transfer or control the flow of thermodynamic energy amongst physical process participants. Each represents a dependency of a rate property (i.e., flow-rate or force) on another dynamical property (i.e., rate or state) and are classified, as in Table 1, according to how energy is exchanged, stored, or controlled during the process. Whereas RO [39] and Process Specification Language (PSL) Ontology [40] represent the spatiotemporal consequences of physical process such as the fusion, fission of participants, rate dependencies define the underlying thermodynamic forces that drive such changes. For the sake of brevity, only selected OPB:*Rate dependency* classes will be discussed as examples of classes that have, so far, been represented in OPB.

OPB:*Constitutive flow dependency* classes represent dependence of a flow-rate of matter (or charge or energy) on the driving force (fluid pressure) differential that drives blood flow from atrium to ventricle in a heart. A key subclass are the OPB:*Force-driven flow dependencies* that are analogues of Ohm’s law which expresses the dependence electrical current flow-rate on a electrical potential (voltage, a force) difference across an electrically conductive pathway. For an “ideal” conductor, flow-rates are proportional to force differential and can be quantitatively characterized by an electrical resistance proportionality (OPB:*Resistance*;) or its reciprocal, conductance (OPB:*Conductance*). Analogues of Ohm’s law apply to the dependence of the velocity of a moving structure to the viscous force resisting the motion, or the dependence of a chemical reaction flux on the chemical potentials of pools of the reactants [26].

In two cases, flow-rates between participants are formulated to depend not on *force* differences but on differences in the *amount* properties of participants and are represented by OPB:*Amount-driven flow dependencies*. Its subclasses are OPB:*Chemical mass-action rate dependency* and OPB:*Diffusion gradient rate dependency* that represent, respectively, the many classes of chemical mass-action rate laws (e.g., Michaelis-Menten enzyme rate law) and Fick’s diffusion rate law.

OPB:*Coupled flow dependencies* are subclasses of OPB:*Force-driven flow dependency* that represent mechanisms by which energy flows from one participant to another in a manner analogous to an electrical transformer. That is, the product of force times flow-rate for one element is proportional (as a “modulus” parameter) to the product of force times flow-rate of the other. The OPB:*Mechanical transformer dependency* represents participants in a single domain, OPB:*Solid mechanical domain*, in which, for example a biceps muscle tendon moves a weight held in a hand. Other transformer dependencies cross domains. For example, OPB:*Chemo-mechanical transducer dependency* applies to process in which chemical potential energy is transduced into mechanical energy as when ATP is consumed to contract myofibrillar proteins. OPB:*Fluid-mechanical transduction* occurs when the mechanical strain energy of the myocardium is transduced into fluid potential energy of the ventricular blood.

OPB:*Constitutive storage dependencies* (Table 1) represent how kinetic or potential energy is stored by participants in processes. The OPB:*Capacitive storage dependency* represent analogues of Hooke’s law that relates the amount (e.g., mechanical displacement) of a participant to forces acting upon it. For example, OPB:Fluid capacitive dependency represents how the fluid pressure (OPB:*Fluid pressure*) of a portion of ventricular blood depends on the amount (OPB:*Fluid amount*) of blood in the ventricle. The more blood, the more fluid potential energy is associated with the blood portion. OPB:*Inductive flow dependencies* represent how elements that behave as analogues of electrical inductors store kinetic energy. For example, OPB:*Fluid inductive dependency* represents the dependency of the momentum of a moving portion of fluid on the fluid pressures that move the fluid just as OPB:*Mechanical inductive dependency* relates the solid momentum of a solid object to the forces that accelerate it.

OPB:*Transactor dependency* classes represent a broad and pervasive class of dependencies for which the actual thermodynamic forces are either unknown or negligible. They are dynamical “wild cards” for representing dependencies that are known to exist but whose mechanisms are unknown, are too complex, or are thermodynamically negligible. Thus, the baroreceptor reflex, the neural control of heart rate and contractility by aortic blood pressure, which involves complex neural signal transmission and transduction is often modelled as a simple proportional dependency. Virtually any combination of dynamical property dependencies can and have been modeled by such simple “A affects B” formulations.

Next, we briefly discuss the “constitutive” properties of rate dependencies that are subclasses of OPB:*Physical property*.

### Constitutive properties of rate dependencies

OPB:*Rate dependencies* are “constitutive” in that the functional form of the dependency depends on both the structural and material constitution of the device described by the dependency. For example, the slope of a dependency between voltage differential and current for a wire depends on structural composition (length, diameter) and material composition (the resistivity of the conducting material). Similarly, vascular blood flow driven by a pressure gradient is a more complex dependency between vessel’s length, diameter, and wall thickness, the wall’s material elastance properties, and the blood’s viscosity. We have introduced such biophysical rate dependencies as analogues of “ideal” electrical circuit elements—e.g., resistors, capacitors, inductors, transformers—whose operating characteristics are simple proportionalities between the values of physical property players. For such ideal dependencies, we use the OPB:*hasConstitutiveProportionality* object property to link to subclasses of OPB:*Constitutive proportionality*, a kind of OPB:*Physical property*, that includes OPB:*Conductance* (along with its reciprocal property, OPB:*Resistance*) and OPB:*Reaction rate constant* (as related to an OPB:*Chemical mass-action rate dependency*).

Biophysicists, however, have to routinely contend with decidedly non-proportional dependencies whose operating curves and algebraic description may entail multiple, empirically-determined coefficients. For example, the resistive flow dependency that relates fluid volume flow-rate to input pressure of a vascular blood flow process is particularly non-linear because the vessel cross-sectional area depends, itself, on input pressure, and because blood is a non-Newtonian fluid whose viscosity depends on flow-rate (see [24]). Furthermore, the conductance of even the simplest membrane ion channel has distinctly non-linear conductive properties that require complex algebraic descriptions that may include dynamical dependencies such as expressed by the Hodgkin-Huxley ion-channel gating equations [25]. Even the familiar Michaelis-Menten rate equation describes a dependency that is non-proportional that requires two coefficients (a half-maximal concentration, and a maximum flow rate) as expressed in SBO. Representing such complexities in broad and complete form is currently beyond the scope of the OPB but will be handled on an as-needed basis as specific use-cases arise.

## Discussion and next steps

The OPB addresses the needs of physiologists for a formal semantics of biophysical processes as needed for the annotation and reuse of biosimulation modeling and data resources [6,8,28]. The OPB schema (Figure 1) extends the classification of continuant and processural spatiotemporal entities (Figure 1, left side; as in BFO, GFO) by introducing (Figure 1, right side) the biophysical abstractions used to quantitatively represent and computationally explain how biological processes occur. We continue to work with collaborators in the biosimulation community to apply and extend OPB and SemSim architectures to the physics-based annotation and analysis of biological processes [6,7]. For such projects we use our SemGen software to parse the code of available biosimulation models to generate a semantic simulation (SemSim) model of each source model based on OPB’s class structure and relational schema. We are able to assemble sets of SemSim models of into an aggregate network knowledgebase (tentatively termed “SemPhysKB” [5]). In preliminary work, we have abstracted the contents of such a knowledgebase into a network of physical entities (as nodes) and the processes (as linking arrows) that we call “PhysioMaps” [10,11] that we propose to analyze with quantitative modeling and qualitative inferencing as in our prior Chalkboard application [41]).

The OPB representational framework is based on general theories of classical physics and network thermodynamics [36,37] as represented in bond-graph theory [35] and the PhysSys ontology [34]. We have exploited this generality to provide an integrated representation of the physical properties and principles that apply to the broad spatiotemporal scales and multiple biophysical domains that are required for quantitative analysis of the “physiome” [27]. Whereas we recognize that OPB could be generalized from discrete to continuum physics, we are deferring such a generalization pending use-cases in the modeling community for which continuum models can accelerate and facilitate continuum modeling projects.

A challenge we faced was to develop a representation of dynamical properties [3] and dependencies that is both orthogonal to and consistent with existing spatiotemporal representations of biological processes as in BFO and GFO. Given the span and generality of OPB, however, there are bound to be some overlaps. For one example, OPB:*Fluid pressure* maps to PATO:*pressure* in the Phenotype Ontology (PATO [20]). OPB is a complement to other biomedical ontologies and semantic resources as have been reviewed in [42] — OPB generalizes, in both scale and domain, on the chemical kinetic focus of SBO (Systems Biology Ontology) while KiSAO (Kinetic Simulation Algorithm Ontology) describes a variety of simulation algorithms, and TEDDY (Terminology for the Description of Dynamics) offers descriptors of the behaviors of simulation results.

### OPB current limitations, next steps

We are aware of a number of limitations that direct our current OPB research and development. First, whereas we have provided (for the most part) human-readable definitions to the new dependency classes, our OWL description-logic implementation of physical dependency classes is far from complete.

A next step is to implement *has_property_player* relations for dependencies (analogous to *has_participant* relations for processes) that link property instances of physical entities according to the particular mathematical role they play the dependency computation. For example, calculating fluid flow-rate from a portion-of-fluid-A to portion-of-fluid-B holds only for the fluid pressure of A, the fluid pressure of B, and the fluid flow resistance from A-to-B; other pressures and resistances are irrelevant. Second, our current collaborations amongst the biosimulation community aim to test the generality, utility, and applicability of the current OPB class structure and object relations. Another key issue is how to more fully support the non-proportional dependencies across biophysical domains to model non-proportional kinetic rate equations (as in SBO) for modeling chemical mass-action kinetics.

## Competing interests

The authors have no competing interests in this work.

## Authors’ contributions

All coauthors participated in numerous discussions leading to the development of the OPB. DLC is the developer of the ontology and led the writing of this manuscript. JHG, FLB, and MLN contributed to the writing and editing of the manuscript. All authors read and approved the final manuscript.
